# Supplementary material for: Autophagy Related Gene (ATG3) is a Key Regulator for Cell Growth, Development, and Virulence of Fusarium oxysporum
Source: Genes (Basel). 2019 Aug 28;10(9):658. doi: 10.3390/genes10090658 (PMC6769740; doi:10.3390/genes10090658)
Supplement: Supplementary file 1 [file genes-10-00658-s001.pdf]

Supplementary Material

Fig.S1

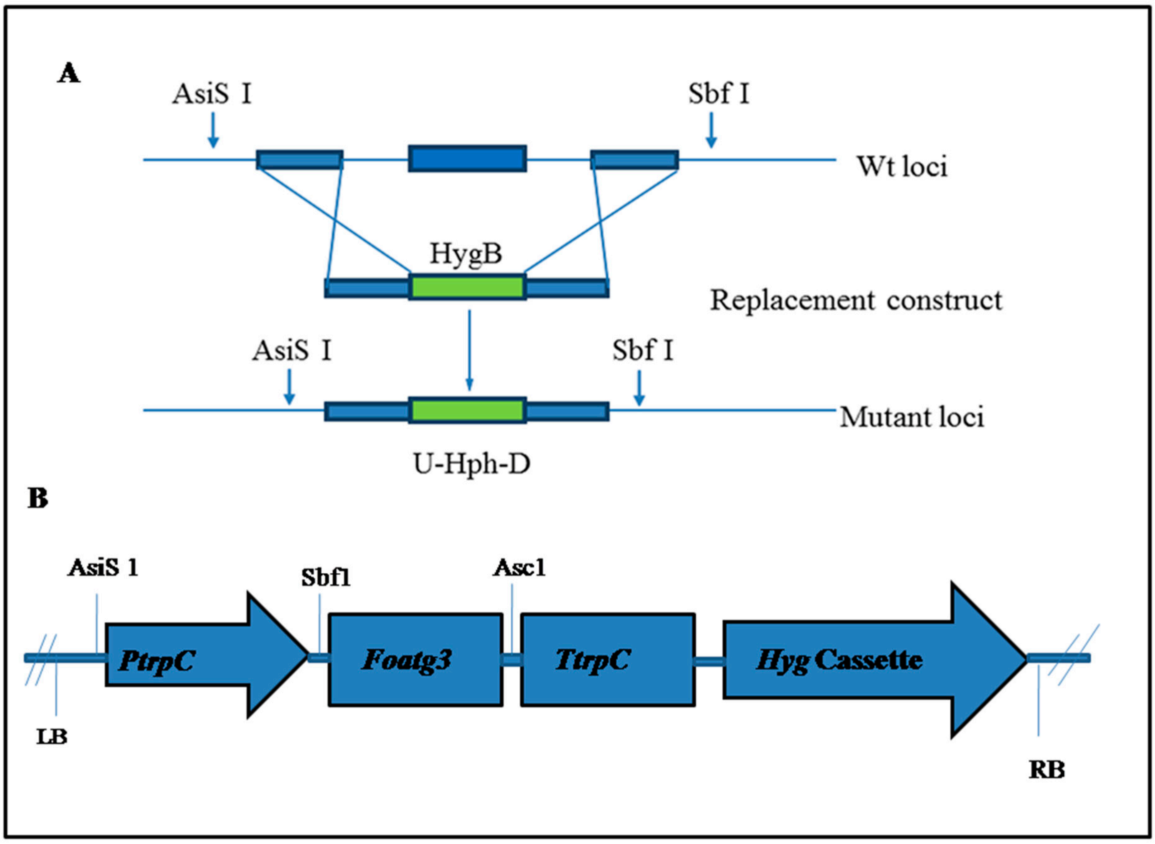

Fig.S1. Image represents map of vectors (A) The Foatg3 coding region was replaced with the HygB cassette. (B) Construction of the GFP Foatg3 fusion protein. The Foatg3 cDNA fragment was amplified with the indicated primers containing Sbf1 and Asc1 restriction sites inserted at 5' and 3' end respectively.

Fig. S2

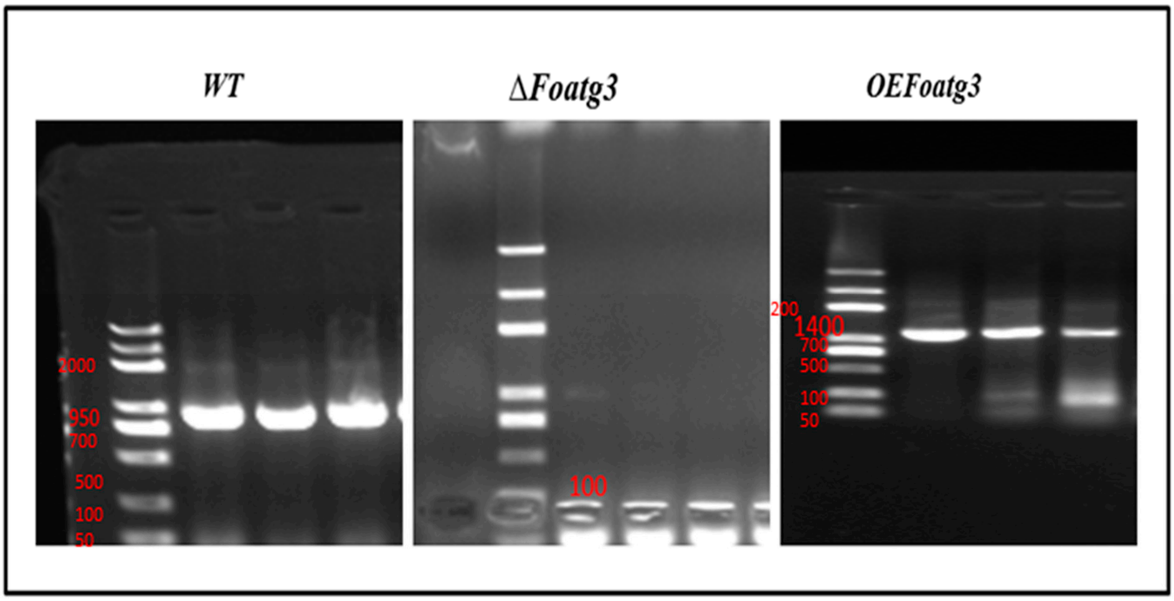

Fig.S2. Image represent expected shift in deletion mutant and over expression of mutants.

Fig.S3

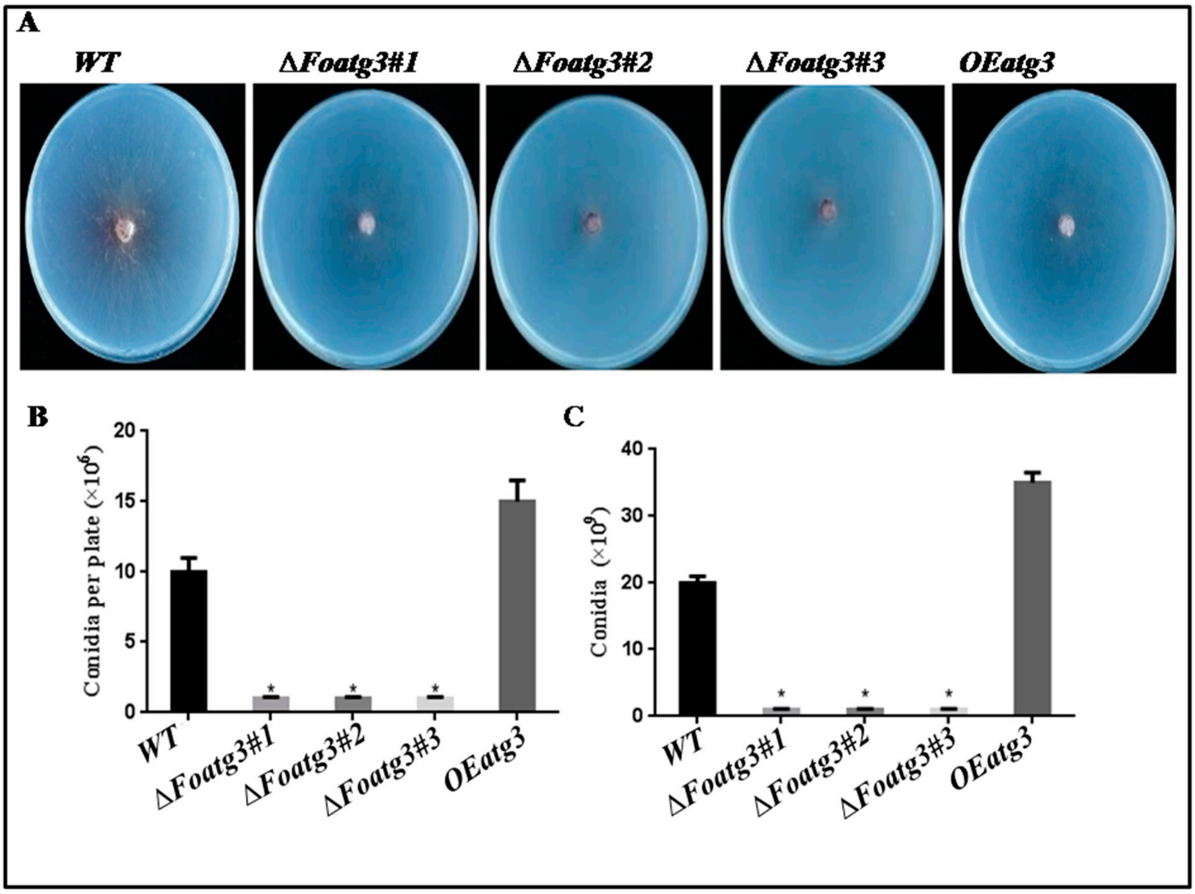

Fig.S3. In nutrient lacking media hyphal formation was very faint. (**A**) Represents the image of 7 days old strains after inoculation. (**B)** After 7 days number of micro conidia recovered from PDA plates which were grown on 28°C. (**C**) Represents the number of recovered micro conidia from 2 days old culture with shaking at 28°C. Bars indicating slandered error from replications. To perform statistical analysis t-test was used. *P <0.05.

Fig. S4

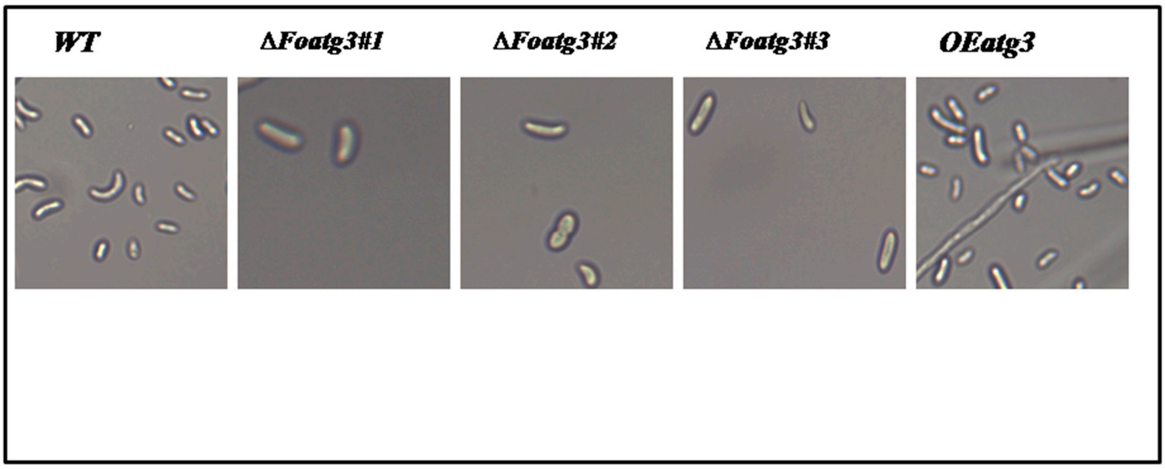

Fig. S4. After seven days of inoculation all strains produced micro conidia. In Foatg3Δ mutant conidial production was suppressed compared to wild type (WT) and overexpression (OE). Image represents conidial germination in different strains.

Table. S1. Primers used for construction of replacement cassette and genes cloning in this study.

Primer name	Primer sequence (5'-3')
**Deletion primers**	
FoATG3 5'-U F (P1)	GCGATCGCGAAGAAGAACCTAGGAGGTACCATCATCGCGAGC

FoATG3 5'-U R (P2)	TGAGTTCAGGCTTTTTCATGGTGGCTGGCTTCGTTTCGTCTCGGTTC
FoATG3-Hph-F(P3)	GAACCGAGACGAAACGAAGCCAGCCACCATGAAAAAGCCTGAACTCA
FoATG3-Hph-F (P4)	AGGCATACTGTAGATACCCTATTCCTTTGCCCTCGG
FoATG3 3'-D F (P5)	CCGAGGGCAAAGGAATAGGGTATCTACAGTATGCCT
FoATG33'-D R (P6)	CCTGCAGGGATAGTGACGGCGAAGGCAGTGGCAAGGC
**Cloning Primer**	
FoATG3 F	CCTGCAGGATGAATTACATCTACTCCACAGTCAACA
FoATG3 R	GGCGCGCCTTAGACACCCATGGTGAAATCGTGC
**qPCR Primer**	
FoATG3F	TACACCCGTCTCTCACAAGTC
FOATG3 R	GAAGATGGCTCACTCTCTGC
**Actin Primers**	
Actin F	TGGTCGTCCTCGTCACACT
Actin R	TGTGCCTCATCCCCAACATA
